# Long-lasting effects of lavender exposure on brain resting-state networks in healthy women

**DOI:** 10.3389/fnins.2025.1555922

**Published:** 2025-06-10

**Authors:** Ron Kupers, Océane Dousteyssier, Jérôme Delforge, Vanessa Gonnot, Kevin Kantono, Bernard Blerot, Arnaud Pêtre, Laurence Dricot, Armin Heinecke

**Affiliations:** ^1^Institute of Neuroscience (IoNS), UCLouvain, Brussels, Belgium; ^2^Department of Neuroscience, Panum Institute, University of Copenhagen, Copenhagen, Denmark; ^3^Brain Impact, Consumer Neuroscience, Auderghem, Belgium; ^4^LMR Naturals By IFF, Grasse, France; ^5^IFF, Hilversum, Netherlands; ^6^NIRx Medizintechnik Gmbh, Berlin, Germany

**Keywords:** olfactory connectome, resting state functional MRI (rsfMRI), salience network (SAL), default mode network (DMN), Independent Component Analysis (ICA)

## Abstract

**Introduction:**

Most brain imaging studies on olfaction focus on short-term odorant stimuli, with few examining long-lasting odor exposure or its after-effects. In this study, we utilized resting-state fMRI (rsfMRI) to investigate the effects of prolonged odor exposure to lavender on brain activity and whether these persist post-exposure.

**Methods:**

Fourteen healthy women underwent two fMRI sessions, conducted one week apart, in a randomized order. Both sessions included rsfMRI scans before, during, and up to 2 h after a 14 min exposure to either lavender essential oil or a non-odorant control.

**Results:**

An Independent Component Analysis identified the salience network (SAL) and default mode network (DMN) as the most consistent resting-state networks. A two-factorial ANOVA revealed significant time-varying interaction effects between the SAL and DMN. During odor exposure, functional connectivity (FC) increased within the SAL, and a negative correlation between the SAL and DMN appeared, which intensified immediately after exposure. Two hours post-exposure, the FC between SAL and DMN turned positive.

**Discussion:**

These findings suggest that prolonged odorant exposure to lavender can induce long-lasting brain effects detectable up to 2 h afterwards in women. This proof-of-concept study should be extended to other odorants and to men, and offers new possibilities for exploring the effects of aromatherapy or other odor exposure interventions on brain activity.

## Introduction

There is now ample evidence that exposure to an odorant is associated with increased activity in a number of brain areas (Arnold et al., 2020; Torske et al., 2022 for recent meta-analyses). These include the piriform cortex, amygdala, entorhinal cortex, anterior and posterior insula, medial and lateral orbitofrontal cortex, anterior and posterior cingulate cortex, and mediodorsal thalamus. Nearly all brain imaging studies on olfaction have studied the response to short olfactory stimuli in the order of seconds, typically between three and six. Although animal studies have examined the effects of long-lasting odor exposure on behavior, brain structure and function (Buonviso and Chaput, 2000; Buonviso et al., 1998; Hernández-Soto et al., 2022; Mandairon et al., 2006), studies in humans investigating brain responses to longer-lasting tonic forms of olfactory stimulation are lacking. This is surprising since we are often exposed to much longer periods of olfactory stimulation in daily life situations. Examples include scented environments (such as the aroma of a forest, the ocean, a hospital, or a swimming pool), aromatherapy, where an individual is exposed to a specific odor for an extended period (typically between 10 and 60 min) (Her and Cho, 2021), the presence of someone wearing perfume, and more. This prompts the question of how the human brain reacts to a prolonged odorant stimulus.

The effects of an odor on brain function may outlast the actual exposure period (for reviews: Johnson, 2011; Kontaris et al., 2020). To the best of our knowledge, there are no published brain imaging studies examining purported lingering effects after exposure to a tonic odorant. Odors are strongly linked to emotions and memories and can induce involuntary autobiographical memories (de Bruijn and Bender, 2018; Green et al., 2023; Reid et al., 2015). Odors can also induce rapid changes in mood (Alaoui-Ismaili et al., 1997; Chen et al., 2021; Cieri et al., 2023; Dal Bò et al., 2022; Ehrlichman and Halpern, 1988; Chen and Haviland-Jones, 1999; Weber and Heuberger, 2008). The influence of odorants on emotion and memory may be attributed to the partial overlap of brain networks responsible for olfaction, emotion and memory, such as the amygdala, entorhinal cortex, hippocampus and orbitofrontal cortex (Arshamian et al., 2013; Maddock et al., 2001; Rolls, 2015; Saive et al., 2014; Soudry et al.,2011). Odorant input is relayed from the olfactory bulb to forebrain and temporal lobe structures involved in emotional processing (the amygdala, insula and medial prefrontal cortex) and memory formation (entorhinal cortex and hippocampus). Taken together, this suggests that odorants may exert long-lasting effects through influencing emotion and memory-related brain networks (Castellanos et al., 2010). In aromatherapy, it is believed the effects of the odorant stimulation persists beyond the actual exposure period, leading to improved mood (Agatonovic-Kustrin et al., 2020), reduced anxiety (Lehrner et al., 2005; Liao et al., 2021) and pain (Liao et al., 2021; Yang et al., 2024), and improved sleep quality (Zhong et al., 2019) and memory (Yang et al., 2021). The main objective of this proof-of-concept study was to test whether long-term odor exposure produces time-varying changes in brain resting state networks. In order to address this question, we used a within-subject cross-over study design in which we acquired longitudinal resting state functional MRI (rsfMRI) before, during, immediately after, and 1 and 2 h after a 14 min odor exposure, or a non-odorant control.

## Materials and methods

### Subjects

Fourteen women, aged between 21 and 45 years (mean age: 38.5 ± 6.6 years) participated in the study. All participants were right-handed, non-smokers and normosmic, as measured by the Sniffin Sticks test (Hummel et al., 1997) that was administered prior to study inclusion. Individuals with a history of neurological disease or currently taking medication affecting the central nervous system were excluded from participation. We included only women because of their better olfactory performance (Brand and Millot, 2001; Sorokowski et al., 2019) and to reduce intersubject variability (Robinson et al., 2015). Participants received 150€ for their time spent and travel costs. The study was approved by the ethics committee of the University Hospital St Luc, Woluwe-Saint-Lambert (Ethics approval number No. B403201112591). All methods were performed in accordance with the relevant guidelines and regulations, and participants provided written informed consent before participating in the study.

### MRI procedures

**FIGURE 1:**
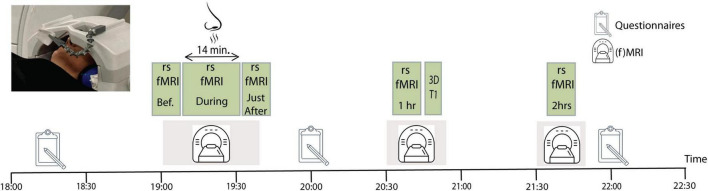
Experimental study design. Each participant went twice through the procedure, once while being exposed to lavender, and once during exposure to a non-odorant substance. The order of the two sessions was counter-balanced across participants. The image to the upper left corner shows the odor administration procedure with the cotton patch attached to the head coil of the magnetic resonance imaging (MRI).

The study protocol consisted of two fMRI sessions which were carried out on different days, spaced 1 week apart, in a randomized order. Half of the participants began with exposure to the active compound, lavender essential oil (IFF, France; CAS: 8000-28-0) at a concentration of 50% in an odorless and non-volatile solvent (isopropyl myristate), while the other half started with exposure to an odorless physiological serum, our control condition. Lavender predominantly activates the olfactory system, but it can also stimulate the trigeminal system, leading to both a scent perception and physical sensations like coolness. The lavender essential oil was administered by placing three drops on a cotton pad positioned on the head coil, 15–20 cm from the participant’s nose inside the MRI room (Figure 1). To avoid that the odorant would spread inside the scanner room, we maintained a constant inverse airflow inside the magnet. The randomized cross-over design allowed each participant to act as their own control. All fMRI sessions took place during the evening, between 6:00 and 10:00 p.m., in order to minimize potential effects due to variations in circadian rhythm.

During the two fMRI sessions, five rsfMRI data sets were acquired: before, during, immediately after, 1 h after and 2 h after odor (or non-odorant control) exposure. All rsfMRI runs lasted 7 min, except the one during odor (or non-odorant control) exposure which lasted 14 min. Participants were instructed to stay awake, have their eyes closed, and to let their thoughts wander freely during scanning. Participants stayed inside the MRI for the first three rsfMRI data acquisitions (Figure 1). Immediately after finishing the odor exposure scan, the experimenter went into the scanner room to remove the cotton pad with the odorant from the head coil, while the participant remained on the scanner table and was repositioned in the magnet. This whole procedure lasted about 3 min, no lingering smell was present in the scanner room by the time the next (immediately after) scan started. After the third rsfMRI session, participants were taken out of the MRI and were guided to a quiet room, where they waited until the next rsfMRI data acquisition. Participants returned to the MRI room for the 1 h after data acquisition, which involved a 7 min resting state scan, followed by a 10 min structural imaging (MPRAGE) acquisition. Thereafter, participants returned to the quiet room where they waited an additional 40 min before returning to the MRI room for the last rsfMRI data acquisition, 2 h after odor exposure. No odor exposure was done in the 1 and 2 h after sessions.

### Behavioral measurements

Upon arrival at the MRI center, participants filled out a circadian typology questionnaire (Horne and Ostberg, 1976) and the Smith Relaxation States Inventory 3 (SRSI) (Smith, 2001) to measure degree of relaxation before the first MRI acquisition. After the third rsfMRI session, participants rated odor intensity on a scale from 0 to 10, with “0” corresponding to not perceptible and “10” very strong intensity. Participants also rated their level of relaxation during the scans on a scale from -10 to 10, with “-10” indicating extremely tense’ and “10” extremely relaxed, and they completed the SRSI for the second time. Following the 2 h after scan, participants completed the SRSI for the third time. Finally, participants rated the likeability of the used odor on a Likert scale from -10 to 10, with “-10” indicating strongly dislike and “10” like it very much.

### MRI data acquisition

Anatomical and rsfMRI scans were performed using a 3 Tesla SIGNA™ Premier GE (General Electric, Milwaukee, United States) MRI Scanner equipped with a 48-channel head coil. The 3D T1-weighted structural images were acquired with the following parameters: a resolution of 1 × 1 × 1 mm^3^, 1 mm slice thickness, repetition time (TR) = 2,187 ms, echo time (TE) = 2.95 ms, flip angle = 8 deg, FOV = 256 × 256 mm^2^. The resting-state sequences consisted of T2*-weighted echo-planar images and were acquired using the following parameters: a voxel size of 2 × 2 × 2 mm^3^, 2 mm slice thickness, TR = 1,700 ms, TE = 30 ms, flip angle = 90 deg, FOV = 220 × 220 mm^2^, 75 slices acquired in an ascending interleaved order. The rsfMRI scans before, immediately after, 1 and 2 h after odor exposition consisted of 253 volumes while the one during odor exposition (or non-odorant control substance) consisted of 497 volumes.

### MRI data pre-processing

The 3D anatomical data was pre-processed as follows. First, we corrected for inhomogeneities in image intensities using a bias field created by the analysis of whiter matter intensity changes over space (Vaughan et al., 2001). This procedure includes an automatic brain extraction step. Next, we normalized the data into MNI space, performing a template matching approach to MNI-152 space represented by the high-resolution template “MNI-ICBM 152 2009c.” The input native anatomy is transformed to the MNI 152 2009c template by minimizing a cost function that reflects the match of the input VMR with the template VMR.

For the functional data, we first performed a slice scan time correction using sinc interpolation, based on information about the TR (1,700 ms) and the order of slice scanning as specified in the original raw data. A 3-D head motion correction was performed to correct for small head movements by spatial alignment of all functional volumes of a subject to the first volume by rigid body transformations. Inspection of estimated translation and rotation parameters revealed that they never exceeded 3 mm or 2°. Drift removal consisted of a linear trend removal, followed by removal of low-frequency non-linear drifts of three or fewer cycles (0.0063 Hz) per time course. For spatial smoothing, we applied a Gaussian filter (FWHM 5 mm) to the volume-based analysis after spatial interpolation to voxel space. Functional data was aligned to the native anatomical data using a two-step procedure. First, we applied position information based on the header of the functional and anatomical scans. Next, we applied a gradient-based alignment to fine-tune the alignment between the two datasets. The functional data was normalized into a four-dimensional representation with 2 × 2 × 2 mm resolution, using the alignment information and the MNI “a12” transformation matrix obtained by the MNI normalization of the anatomical data.

### fMRI analysis

#### Seed-based correlation (SBC) analysis

To define our seed regions-of-interest, we took the coordinates from a meta-analysis on the Default Mode Network (DMN) and the salience network (SAL) to extract the time courses for the SBC analysis (Pievani et al., 2017). For the posterior cingulate cortex (PCC), we drew a sphere of 5 mm radius around the x, y, z coordinates 0, -56, 26, whereas for the right anterior insular cortex, we drew a 5 mm sphere around coordinates 40, 14, -2. This approach allowed us to identify known resting state networks at all time points and conditions. The seed-based correlation approach averages the time course of all functional voxels within the selected region of interest (seed) and performs a pairwise correlation within all functional voxels in the dataset. The method is discussed in comparison to other resting analysis techniques in Seewoo et al. (2021).

#### ICA-based probabilistic maps

We first applied a single-run ICA (Formisano et al., 2002a; Formisano et al., 2002b; Formisano et al., 2004), followed by a Group ICA (Esposito et al., 2005) to all the 140 functional runs (14 subjects, five time points and two sessions) of the sample. The single subject ICA plugin implements methods described in Formisano et al. (2002a),b, 2004 and includes a C++ implementation of the fastICA algorithm (Hyvärinen and Oja, 2000; Esposito et al., 2002). Prior to the ICA decomposition, we performed a principal component analysis (PCA) to reduce the dimensions of the functional dataset from the original number of timepoints to 40, which corresponds to more than 20% of the initial temporal dimensions and accounted for more than 99.9% of the total variance/covariance in all subjects. Next, we applied the self-organizing group ICA (sogICA) procedure to the ICA decompositions obtained from the datasets of each subject, using a C++ plugin in BrainVoyager, according to the methods and component clustering algorithm described in Esposito et al. (2005). In this step, the independent components from the individual datasets are “clustered” at the group level. The clustering algorithm is based on the components’ mutual similarity measures implemented as linear spatial correlation in a common anatomical space. In general, the sogICA framework allows the similarity matrix to be a combination of spatial and temporal measures. Using pure spatial similarity allows investigation of the consistency of the independent components at the group level. The similarity matrix is then transformed into a dissimilarity matrix, which is used as a “spatial distance” matrix within a hierarchical clustering algorithm (see also Himberg et al., 2004). The cluster “group” components were calculated as random effects maps. The random effects statistic for each voxel was calculated as the mean ICA z-value of that voxel across the individual maps divided by its standard error, resulting in a t-statistic, which was converted to a z-statistic.

Next, we performed an analysis of variance (ANOVA) on the sorted components of each group’s ICA result to identify the major resting state networks consistent among subjects, time points and conditions. The identification of network components was performed on the basis of the spatial maps of the ICA components. This procedure allowed us to identify the classical resting state networks (Supplementary Figure 1). Within the selection of detectable resting state networks, we focused on the SAL and the DMN since they were the most similar networks with the highest rank orders within the sorted group components, and identifiable at all time points. After detailed inspection of these two resting state networks, we decided to focus our further analyses on the SAL since it met the following requirements: no difference between lavender and control conditions at “baseline” (before), a significant difference “during.” and a persistent, though smaller, difference during the post-exposure time points (collapsed into a single “After” timepoint; Supplementary Figure 2). Within the DMN, no such temporal pattern was detected. We selected and saved the consistent component map of the SAL of all participants and conditions into a common “volume map” structure, combining all the SALs of all the functional runs. Then, we ran two-factorial ANOVAs on the SALs with group (lavender and control) and time of measurement (before, during, immediately after, 1 after and 2 h after) as independent variables. This approach allows for the analysis of the main effects of “condition” and “time point,” as well as their interaction.

## Results

### Behavioral data

Average odor intensity ratings were 8.4 ± 1.3 in the lavender condition versus 1.8 ± 1.2 in the control condition (*P* < 0.0001), indicating that our odor exposure procedure worked well. Average odor liking ratings were 6.4 ± 3.7 for lavender versus 2.0 ± 3.6 in the control condition (*P* < 0.05), indicating that on average the participants liked the lavender smell. Average relaxation ratings during odorant exposure were not significantly different between conditions and were 6.4 ± 2.6 and 4.4 ± 4.7 for the lavender and control condition, respectively (Table 1). We also did not find a significant difference between the odor and control conditions at any of the measured time points for the degree to which participants succeeded to let their thoughts roam freely (Table 1). No overall effect of odor exposure was found for the SRSI data (Supplementary Figure 3).

**TABLE 1:** Relaxation and roaming thoughts.

	Relaxation		Roaming thoughts	
	Control	Lavender	Control	Lavender
Before	4.4 ± 4.7	6.4 ± 2.6	7.2 ± 1.9	7.6 ± 1.7
Immediately after	5.1 ± 5.0	6.6 ± 2.3	7.6 ± 2.7	7.6 ± 1.5
2 h after	6.0 ± 3.6	5.9 ± 3.0	6.2 ± 3.0	5.6 ± 2.9

Relaxation and roaming thoughts were measured using numerical rating scales (methods).

### MRI data - seed-based correlation (SBC) analysis

**FIGURE 2:**
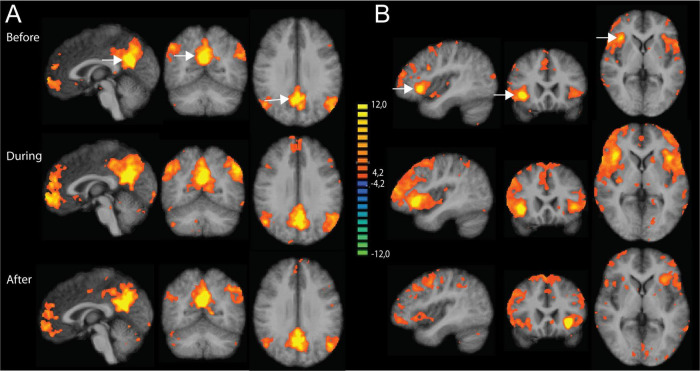
Results of the seed-based correlation (SBC) approach using the posterior cingulate cortex (PCC) and right anterior insula as seed regions. Results are shown for the three time points: before, during and after odor exposure. **(A)** SBC maps based on PCC (x = 0, y = -56, z = 26) as seed region. **(B)** SBC maps based on the right anterior insula (x = 40, y = 14, z = -2) as seed region. All maps are thresholded using the FDR-correction method. The two seed regions are indicated with white arrows in the upper part of the figure.

Figure 2 shows a second level t-map based on the original correlation values for the control conditions at the three time points, using the PCC and right anterior insula as seed regions. As shown, all maps show some correlated regions not belonging to the DMN or SAL networks, suggesting residual noise within the pre-processed data. Subtracting the resulting correlation (r)-values between the odor and the control conditions did not show significant differences for any of the time points. This null effect can be due to the residual noise within the data, masking any potential effects of interest. Therefore, in a next step, we used an Independent Component Analysis (ICA) to properly separate different types of residual noise from effects consistent with the classical resting state networks.

### MRI data - ICA-based analysis: probabilistic map SAL

**FIGURE 3:**
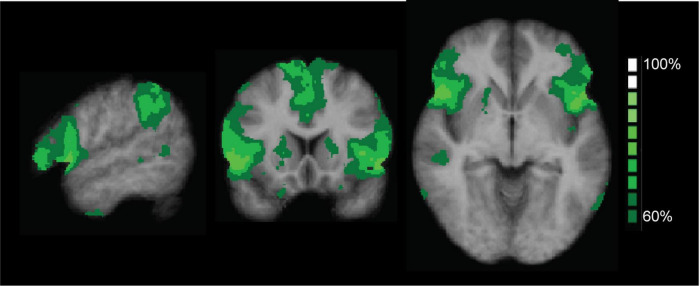
Probabilistic map of the salience network (SAL), thresholded at 60% overlap between subjects, for the two conditions (odor and control) and the five time points. The map confirmed that the proper regions of interest were consistently selected for the conditions and time points. The color coding shows areas of overlap between the different maps.

To check the validity of our ICA approach, we combined the SAL maps of all conditions and all time points into a probabilistic map. Figure 3 shows the consistency between all selected component maps, thresholded at 60% minimal probability. The resulting probabilistic map for the different time points confirmed that the proper regions of interest were consistently selected for the conditions and time points, providing a solid basis for running the ANOVA including the results of the ICA.

### Two-factorial ANOVA of the SAL maps

**FIGURE 4:**
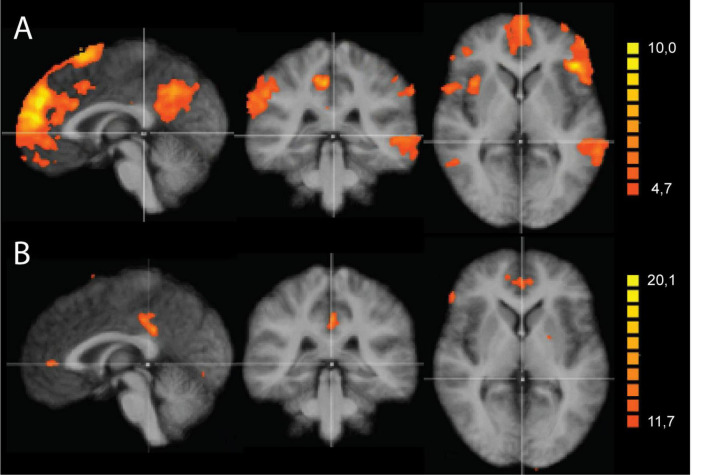
Two-factorial analysis of variance (ANOVA) of the salience network (SAL) maps with the factors odor and time point. **(A)** Main effect of the factor odor, collapsed over all time points. The significant regions coincide with large parts of the default mode network (DMN), suggesting a potential interaction between the SAL and DMN networks. Significant areas included clusters in the posterior cingulate cortex, perigenual anterior cingulate, inferior prefrontal cortex (BA 47) and superior prefrontal cortex (BA 6, BA 8). **(B)** Main effect of the factor time, pooled over the two odor conditions. Two clusters were observed, a large one in the posterior cingulate cortex, just anterior to the cluster observed for the factor odor, and a second one in the perigenual anterior cingulate cortex. Results were thresholded using the cluster threshold correction method. All maps shown at *P* < 0.005.

We first ran a two-factorial ANOVA of the SAL network maps with the factors odor and time point. Results were thresholded using the cluster threshold correction method (Wang and Li, 2015). Figure 4A and Table 2 show the main effect of the factors time, collapsed over the two conditions, and odor, collapsed over all time points. Interestingly, the significant regions coincide with large parts of the DMN, suggesting a potential interaction between the SAL and DMN networks. More specifically, for the factor time, significant areas included one large cluster in the posterior cingulate cortex, a more anterior one in the perigenual anterior cingulate, two in the inferior prefrontal cortex (BA 47) and two in the superior prefrontal cortex (BA 6, BA 8) (Table 2). The ANOVA further showed a significant effect of the factor odor pooled over all the time points, with one large cluster in the posterior cingulate cortex, just anterior to the cluster observed for the factor time, and a second one in the perigenual anterior cingulate cortex (Figure 4B). We calculated the eta-squared value (Maher et al., 2013) as a measure of effect size for the main effects and their interaction. This resulted in the following eta-squared values: 0.0131 for the factor odor, 0.3201 for the factor time and 0.2630 for the interaction of odor and time.

**TABLE 2:** Analysis of variance (ANOVA): main effect of odor.

		MNI coordinates					
Brain area	BA	x	y	z	Volume	F	*P*-value
Posterior cingulate gyrus	BA 31	–3	–32	38	1.096	52.03	0.000007
Supramarginal gyrus	BA 40	–62	–45	30	564	43.72	0.000017
Superior frontal gyrus	BA 6	13	4	70	1.097	41.16	0.000023
	BA 8	–22	34	34	183	20.46	0.000573
Cerebellum	–	42	–72	–45	303	37.13	0.000038
Inferior frontal gyrus	BA 47	–27	31	–23	310	30.73	0.000095
	BA 47	55	32	3	988	28.91	0.000126
Cuneus	BA 17	–13	–108	4	175	30.53	0.000098
Lingual gyrus	BA 18	2	–79	–10	159	24.81	0.000251
Perigenual cingulate	BA 32	–8	46	3	471	23.76	0.000304
Middle frontal gyrus	BA 8	–35	22	44	198	21.55	0.000461
Globus pallidus	–	–22	–1	4	235	19.70	0.000669
	–	–54	21	–8	192	16.26	0.001423

**FIGURE 5:**
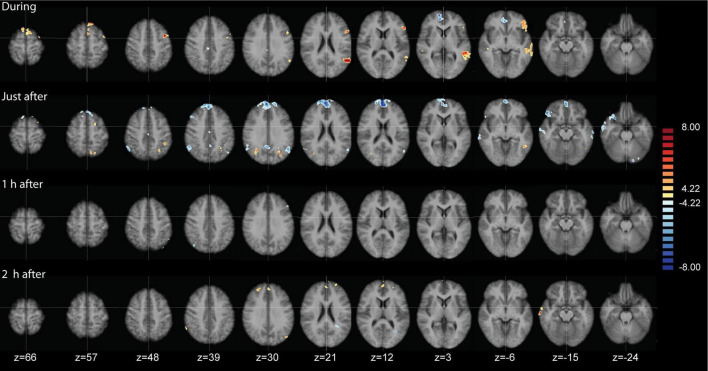
Analysis of variance (ANOVA) results of the functional connectivity maps of the salience network (SAL), contrasting lavender with no odorant control (Lavender > Control) at four time points. Data are presented on axial slices. The numbers at the bottom refer to the z-coordinates in MNI space. Images are presented in radiological convention (left part of the brain is shown to the right). Cluster correction was applied to correct the maps. All clusters shown are at q(FDR) < 0.001.

While the above-described ANOVA is the best approach to identify main effects and interactions between factors, any specific *post hoc* contrast between pairs of conditions will be biased by the inclusion of all the conditions within the error term applied. To be able to calculate a statistically more correct error term, we ran additional more simplified ANOVA models, including only two levels of the time factor. In total, we performed four 2 × 2 ANOVAS including the two conditions and two time points: during and before, immediately after and before, 1 h after and before, and 2 h after and before. Figure 5 shows the results of the interaction effects at the different time points. During odor presentation, we observed several clusters of increased and decreased FC with the SAL network. More specifically, clusters of increased FC with the SAL were found in bilateral middle temporal gyrus (BA 21), bilateral superior frontal gyrus (BA 6, BA 8), left middle frontal gyrus (BA 6), left inferior frontal gyrus (BA 45) and right temporo-parietal junction (BA 39) (Figure 5 and Table 3). In addition, there were two clusters of decreased FC, one in the right perigenual cingulum (BA 32) and the other in the right posterior cingulate (BA 31). Both these areas are part of the DMN. In the immediately after condition, he FC of the SAL changed substantially, with a majority of areas with decreased FC with the SAL, especially brain areas that are part of the DMN (Figure 5 and Table 4). More specifically, a reduction in FC with the SAL network was found in the right middle temporal gyrus (BA 21), bilateral supramarginal gyrus (BA 39), right inferior frontal gyrus (BA 45, BA 47), right middle frontal gyrus (BA 8), right dorsal frontal gyrus (BA 10), precuneus (BA 7), cingulate gyrus (BA 24), left inferior temporal gyrus (BA 20) and cerebellum. A smaller number of brain areas showed increased FC with the SAL network, including bilateral angular gyrus (BA 39), left superior parietal lobule (BA 7), left superior frontal gyrus (BA 6), left inferior parietal lobule (BA 40) and left inferior temporal gyrus (BA 37). At 1 h post odor exposure, there were four small clusters of decreased FC with the SAL network, including the right angular gyrus (BA 39), left inferior (BA 7) and left superior (BA 7) parietal lobule, and left middle frontal gyrus (BA 46) (Figure 5 and Table 5). Interestingly, at 2 h post exposure, clusters of increased FC emerged, several of them belonging to the DMN. These clusters included the right supramarginal gyrus (BA 40) and the right middle and superior frontal gyri (BA 9). Additional increases in FC were found in right middle temporal gyrus (BA 21), left middle occipital gyrus (BA 19) and right cerebellum (Figure 5 and Table 6). We calculated Cohen’s d (Lakens, 2013) as a measure of effect size for the *post hoc* contrasts described above. We found an average Cohen’s d of 0.952 for the regions showing a positive difference between lavender and control odors, while Cohen’s d for the regions showing a negative difference was -2.162.

**TABLE 3:** 2-way analysis of variance (ANOVA) (odor > control): during.

		MNI coordinates					
Anatomical region	BA	x	y	z	t	*P*-value	Nr voxels
**Increases in FC**							
Middle temporal gyrus	BA 21	46	–32	–1	6.45	0.000022	654
	BA 21	–49	–36	0	8.14	0.000002	6254
Superior frontal gyrus	BA 8	10	27	66	5.87	0.000055	484
	BA 6	–5	15	72	7.95	0.000002	2235
	BA 8	–9	42	56	7.35	0.000006	926
Middle frontal gyrus	BA 6	–42	6	47	9.18	0.000000	2505
Inferor frontal gyrus	BA 45	–55	26	12	7.05	0.000009	3386
	BA 44	–52	15	25	7.46	0.000005	1056
Temporo-parietal junction	BA 39	–50	–59	23	10.74	0.000000	2477
**Decreases in FC**							
Perigenual cingulate gyrus	BA 32	9	51	–1	–11.82	0.000000	3311
Posterior cingulate gyrus	BA 31	7	–26	34	–6.82	0.000012	797

**TABLE 4:** 2-way analysis of variance (ANOVA) (odor > control): immediately after.

		MNI coordinates					
Anatomical region	BA	x	y	z	t	*P*-value	Nr voxels
**Increases in FC**							
Angular gyrus	BA 39	32	–70	21	6.76	0.000013	924
	BA 39	–31	–76	33	7.83	0.000003	1,673
Superior parietal loblue	BA 7	–9	–67	55	5.98	0.000046	408
Superior frontal gyrus	BA 6	–23	10	60	7.14	0.000008	812
Inferior parietal lobule	BA 40	–42	–45	51	8.03	0.000002	546
**Decreases in FC**							
Dorsal frontal gyrus	BA 10	4	61	8	–13.31	0.000000	24,553
Cerebellum		28	–82	–33	–10.49	0.000000	1,335
		–21	–82	–40	–8.26	0.000002	3,389
Inferior frontal gyrus	BA 47	43	21	–19	–8.66	0.000001	3290
	BA 45	56	21	6	–6.77	0.000013	669
Middle temporal gyrus	BA 21	59	1	–33	–8.23	0.000002	4,296
	BA 21	–70	–16	–16	–7.27	0.000006	542
	BA 21	68	–31	–4	–6.73	0.000014	721
Supramarginal gyrus	BA 39	55	–61	36	–7.73	0.000003	3,181
	BA 40	–52	–65	33	–6.85	0.000012	2,470
Middle frontal gyrus	BA 8	44	24	38	–6.79	0.000013	414
Precuneus	BA 7	1	–55	31	–6.92	0.000011	1,287
Cingulate gyrus	BA 24	–1	–15	37	–6.31	0.000027	328
Inferior temporal gyrus	BA 37	–48	–55	–8	6.86	0.000012	542
	BA 20	–53	1	–34	–6.39	0.000024	437

**TABLE 5:** 2-way analysis of variance (ANOVA) (odor > control): 1 h after.

		MNI coordinates					
Anatomical region	BA	x	y	z	t	*P*-value	Nr voxels
**Decreases in FC**							
Angular gyrus	BA 39	40	–75	37	–7.15	0.000007	186
Inferior parietal lobule	BA 7	–37	–81	47	–7.02	0.000009	192
Superior parietal lobule	BA 7	–51	–63	51	–5.08	0.000212	190
Middle frontal gyrus	BA 46	–51	29	31	–6.20	0.000032	174

**TABLE 6:** 2-way analysis of variance (ANOVA) (odor > control): 2 h after.

		MNI coordinates					
Anatomical region	BA	x	y	z	t	*P*-value	Nr voxels
**Increases in FC**							
Middle temporal gyrus	BA 21	68	–14	–14	10.04	0.000000	370
	BA 21	64	2	–11	6.05	0.000041	313
Superior frontal gyrus	BA 10	–26	53	16	7.34	0.000006	439
	BA 9	0	56	12	5.78	0.000064	1484
Supramarginal gyrus	BA 40	62	–54	39	5.04	0.000225	291
Middle frontal gyrus	BA 9	24	47	30	7.07	0.000008	385
Middle occipital gyrus	BA 19	–46	–77	30	6.10	0.000038	280
Cerebellum		27	–80	–29	5.71	0.000072	380
**Decreases in FC**							
White matter		–32	–63	13	–7.72	0.000003	233
		–26	–49	20	–6.21	0.000032	219

We also checked how odor exposure affected the rankings of the DMN and SAL ICA group components across the different time points. As shown in Table 7, before odor exposure, the SAL ranked as components 12 and 11 for the control and lavender conditions, respectively, During odor exposure, the SAL moved up to rank six for the lavender condition and up to rank seven in the control condition. The latter observation could be due to odor expectation. In the immediately after condition, the SAL network ranked fifth place in the lavender condition compared to 14^th^ place in the control condition. At 1 h after, the SAL still ranked sixth place in the lavender condition, to move down to eighth place at 2 h after. In line with the expectations, the DMN ranked higher (third) at the before time point for the two conditions. In the lavender condition, the DMN moved to the fifth place at the during and immediately after time points, and further up to the fourth place at 2 h. In the control condition, the DMN ranking stayed very consistent, except in the during condition where it moved up to the fourth place.

**TABLE 7:** Ranking of salience network (SAL) and default mode network (DMN) Independent Component Analysis (ICA) components at all time points.

	SN		DMN	
Time point	Control	Odor	Control	Odor
Before	12	11	3	3
During	7	6	4	5
Just after	14	5	3	5
1 h after	7	6	2	3
2 h After	10	8	3	4

To test for the potential bias of the double duration of the “during” scan, we ran another two-factorial ANOVA in which we excluded the “during” time point. This analysis resulted in very similar effects, indicating that the influence of the during time point on the interaction effect detected is negligible (Supplementary Figure 4).

## Discussion

We used rsfMRI to study the long-lasting effects of an odorant on brain activity. Participants passively inhaled lavender oil for 14 min and its effects on brain resting state networks was investigated up to 2 h after exposure. An ICA showed that the SAL and DMN were the most robust and consistent resting state networks identified throughout the 2 h period. During lavender exposure, activity in the SAL increased compared to before. Using the SAL maps, a two-way ANOVA with the factors “odor” and “time” showed significant interaction effects between the SAL and DMN networks which evolved over time, but persisted up to 2 h after. These data provide the first evidence that effects of an odorant can still be measured 2 h after exposure.

We employed a novel method of continuous odor exposure that has not previously been used in brain imaging studies of odor perception. Nearly all previous studies have measured brain responses to short phasic odorant stimuli, lasting typically in the order of seconds. In this study, we presented an odorant for 14 min. Also different from most brain imaging studies on olfaction, we did not use an olfactometer or an airflow-based delivery system. Instead, we used a more natural method that consisted of presenting the odorant by placing a piece of tissue that was impregnated with the lavender solution to the MRI head coil. This was done to mimic as close as possible a condition resembling aromatherapy in which passively an odorant is inhaled over a longer period. Our odor exposure method worked well as evidenced by the average odor intensity and pleasantness ratings. Also new is that we measured the effects on brain activity up to two hours after the odorant source was removed. Finally, we emphasize that participants served as their own controls since they were all scanned during an odorant and a non-odorant condition.

Odorants can be of three types: pure olfactory, trigeminal and mixed olfactory-trigeminal, Although lavender most strongly activates the olfactory system, it can also activate the trigeminal system, particularly when used as an essential oil or in high concentrations. Pure olfactory odorants activate the olfactory nerve (cranial nerve I) which transmits signals from the olfactory epithelium in the nasal cavity to the brain’s olfactory bulb (Brand, 2006; Schaefer et al., 2002). These odorants are experienced solely as a smell, without accompanying sensations such as coolness or irritation (Brand, 2006). In contrast, trigeminal odorants stimulate both the olfactory nerve and the trigeminal nerve (cranial nerve V), which is responsible for conveying sensations like burning, cooling, tingling, stinging, or irritation from the nasal and oral cavities (Brand, 2006; Frasnelli et al., 2011). As a result, trigeminal odorants are perceived not only as a smell but also as a physical sensation in the nose or throat. It is worthwhile mentioning that lavender essential oils also exert anxiolytic (Agatonovic-Kustrin et al., 2020) and analgesic effects (You et al., 2024).

During odor exposure, the most conspicuous change was a reduction in FC of the SAL with the posterior cingulate and perigenual cingulate cortices, two important key nodes of the DMN. In addition, there were also noticeable increases in FC within the SAL, indicating increased involvement of the SAL during odor exposure. Together, these findings suggest an increased engagement of the SAL and an inhibitory effect on the DMN during odor exposure. Of interest, increased FC within the SAL was shown in studies of the effects of mindfulness training (Bremer et al., 2022). FC of the SAL with the right middle temporal gyrus and the left temporo-parietal junction (TPJ) also increased during odor exposure. These areas are part of the social brain network (Amft et al., 2015; Pitcher and Ungerleider, 2021). The TPJ is functionally connected with the SAL (Amft et al., 2015; Pitcher and Ungerleider, 2021) and is implicated in attention and awareness, particularly in the context of directing attention to relevant stimuli in the environment and monitoring changes in one’s surroundings. The increased FC of the TPJ with the SAL in our study could reflect the response to a change in the odorant environment. The TPJ is also involved in various social cognitive functions, including theory of mind, empathy, moral reasoning and perspective-taking (Saxe, 2006; Schurz et al., 2017).

In the immediately after condition, negative correlations of the SAL with other brain areas dominated, especially with those belonging to the DMN. At this time point, we also observed a strong positive correlation between the SAL and the angular gyrus. This brain area plays a critical role in cortical speech and language processing, in memory retrieval, and in the integration of information involving sensory modalities for semantic processing (Seghier, 2013). The negative FC between the SAL and DMN was no longer present 1 h after. However, at 2 h post odor exposure, a positive FC between the two networks emerged. Taken together, this suggests a time-resolved dynamic interaction between the SAL and DMN networks which is initially negative, reaches its maximum effect immediately after the odor presentation and changes into a positive correlation 2 h after. A positive FC between the SAL and DMN was shown in other conditions such as mindfulness meditation (Bremer et al., 2022) and social cognition (Ribeiro da Costa et al., 2022). It may therefore be tempting to speculate that one of the long-term effects of tonic lavender oil exposure is its capacity to couple brain activity in key nodes of the SAL and DMN in an adaptive manner. Our findings can also be reconciled with the default-mode interference hypothesis (Sonuga-Barke and Castellanos, 2007) which states that DMN activity can persist or remerge during goal-directed tasks, allowing it to compete with task-specific neural processing.

What are the specific roles of the SAL and DMN in perception and human cognition? A large body of evidence suggests that the SAL is primarily involved in detecting and filtering important or salient stimuli from the environment and internally generated thoughts (Seeley et al., 2007; Menon, 2015, 2023). The SAL helps in directing attention to relevant stimuli and in coordinating appropriate responses. Key regions of the SAL network include the anterior insula and the anterior cingulate cortex (Seeley et al., 2007; Menon, 2015). The anterior insula is involved in processing bodily sensations and emotional experiences, while the anterior cingulate cortex is associated with monitoring of conflicts and errors. The anterior insula is also involved in odor discrimination (Plailly et al., 2007) and forms a key node of the olfactory connectome (Arnold et al., 2020). Subcortical components of the SAL are the ventral striatum, amygdala and the substantia nigra/ventral tegmental area (Seeley et al., 2007; Menon, 2015). The SAL network integrates sensory, cognitive and emotional information and thus contributes to multiple complex brain functions such as communication, social behavior and self-awareness (Menon and Uddin, 2010; Feng et al., 2021; Craig, 2002, 2010). In contrast, the DMN is active when at rest or engaged in internally focused tasks such as daydreaming, mind-wandering, or self-referential thinking. Key regions of the DMN include the medial prefrontal cortex, posterior cingulate cortex, and inferior parietal lobule. The DMN is involved in processes related to self-referential thinking, autobiographical memory retrieval, social cognition, and theory of mind (Raichle, 2015; Smallwood et al., 2021). The SAL and DMN interact dynamically to facilitate adaptive behavior. When a salient stimulus or task-relevant information is detected, the SAL becomes activated, directing attention toward it and suppressing activity in the DMN. Conversely, during internally focused tasks or when the external environment is less salient, the DMN becomes more active, while the SAL decreases its activity (Sridharan et al., 2008). The interaction between these networks allows for the flexible allocation of cognitive resources depending on the situational demands, ensuring efficient cognitive processing and behavior regulation. Dysregulation or imbalance in the interaction between these networks may contribute to cognitive and emotional disturbances observed in various neuropsychiatric disorders (Menon, 2011). Several nodes of the DMN overlap with brain regions involved in social and affective processing. These areas include the posterior cingulate. TPJ, ventromedial prefrontal cortex, middle temporal gyrus, subgenual cingulate cortex and precuneus. This has been called the extended social-affective default network (Amft et al., 2015). Many of these brain areas have odor-induced alterations in FC with the SAL, suggesting dynamic interactions between detecting and filtering important or salient stimuli from the environment and social and affective processing.

The evolutionary significance of prolonged odor exposure may be linked to survival, social and reproductive communication, and environmental adaptation. Certain odors, such as those from spoiled food, smoke, or chemical threats, serve as danger signals (Mutic et al., 2017). Even with prolonged exposure, it is crucial that particularly strong or harmful odors remain detectable to maintain vigilance and facilitate avoidance (Ferdenzi et al., 2014). Across many species, including humans, body odors and pheromones play a role in social bonding, mate selection, and group recognition (Bakker et al., 2021 for a recent review). Extended exposure to these social odors may strengthen relationships or indicate reproductive status (Lübke and Pause, 2015). Additionally, the olfactory system has strong connections to the limbic system, which regulates emotions and memory (Kontaris et al., 2020). Prolonged exposure to specific odors may reinforce associative learning by linking a scent to safety, comfort, or past experiences. Prolonged odor exposure can also affect mood, stress levels, and cognitive function. For example, soothing scents like lavender may encourage relaxation (López et al., 2017), while unpleasant or irritating odors can heighten alertness or trigger avoidance responses (Iravani et al., 2021). Additionally, long-term odor exposure is essential for environmental awareness and adaptation, as both humans and animals must continuously monitor their surroundings to respond effectively to changes. The ability to detect and adapt to persistent odors (e.g., the scent of food or water) can enhance survival in changing environments. Finally, long-term odor exposure may lead to habituation and sensory adaptation, whereby where the brain reduces its response to a constant stimulus (Pellegrino et al., 2017). Habituation helps organisms focus on new, potentially more important smells (e.g., detecting a predator or food source in a familiar environment) (Mignot et al., 2022).

This study has a number of limiting factors. First, we only included 14 subjects which is at the low end in fMRI studies. However, the fact that subjects served as their own control and were tested twice under very similar conditions partly compensates for the smaller sample size. Another limiting factor is that we tested a single odorant, a single exposure duration and a single odor concentration. It is important to test for various concentrations in olfactory neuroscience (Wachowiak et al., 2025). Considering that each participant already underwent 10 resting state scans, spread out over two different sessions, testing for the effects of different odorants, concentrations and exposure times seemed very difficult. For this study, we selected lavender, which belongs to the aromatic and floral odor families. Lavender is characterized by fresh, herbal, and slightly sweet notes and is widely used in aromatherapy, as well as in many feminine fragrances. The odorant stimulus was presented for 14 min, a prolonged exposure that may have contributed to physiological habituation to the scent and potential shifts in attention. However, we did not control for the influence of these factors on our results, as doing so would have required behavioral measurements during fMRI data acquisition—an approach we opted against to maintain the resting-state nature of the study. We emphasize that incorporating an attentional task was not our intention. Participants were not instructed to focus on the stimuli; they were simply asked to close their eyes and let their thoughts flow freely during MRI data acquisition. Future studies could assess whether the same results are obtained when subjects are instructed to pay attention to the odorant stimulus or when imposing an odor-related task. Additionally, we did not measure fluctuations in perceived odor intensity and quality over time. However, we assume that evaporation likely influenced these aspects. The odor intensity of lavender oil on a cotton pad follows a typical evaporation curve, initially strong but gradually diminishing as the most volatile compounds dissipate over time. This process may also lead to perceptual shifts, transitioning from fresh, floral, and slightly citrusy top notes to muskier, woody, or earthy undertones by the end of the 14 min exposure. Another limitation of our study is that we included only women, making it necessary for future research to determine whether the findings can be replicated in a male population. It would have been interesting to measure also the menstrual status at the moment of testing. However, our ethics approval did not allow to acquire information on the menstrual cycle. Our study should therefore be considered a proof of concept, demonstrating that prolonged exposure to a tonic odorant can induce lasting effects on brain activity. Future research should investigate whether these findings extend to other odor families or scents with different emotional valence or social significance. Indeed, there is evidence that emotions induced by smells impact resting state functional brain connectivity in a valence-specific manner (Carlson et al., 2020). Additionally, a systematic exploration of the impact of odor exposure duration and concentration is needed. For instance, unpleasant or socially relevant odors, such as those associated with the human body, can trigger strong emotional and attentional responses and may produce time-dependent effects that differ from those observed in this study (Calvi et al., 2020; Rolls et al., 2003).

In conclusion, we used resting state fMRI to study the long-lasting effects of the exposure of a positively-valenced odor on the interaction of large-scale brain networks. Our data show time-resolved dynamic interactions between the SAL and the DMN that could be measured up to two hours following odor exposure. Of notice is the positive functional connectivity between the SAL and DMN that was measured after 2 h and that may suggest a coupling of brain activity within SAL and DMN in an adaptive manner.

## Data Availability

The raw data supporting the conclusions of this article will be made available by the authors, without undue reservation.
