# Supplementary material for: Long-lasting effects of lavender exposure on brain resting-state networks in healthy women
Source: Front Neurosci. 2025 Jun 10;19:1555922. doi: 10.3389/fnins.2025.1555922 (PMC12186306; doi:10.3389/fnins.2025.1555922)
Supplement: Supplementary file 1 [file Supplementary_file_1.pdf]

Supplementary Information

Lavender oil composition

The lavender oil used in this study is characterized by the following specifications: density D20/4 0.874-0.890, refraction index at 20°C 1.459-1.464, optical rotatory power at 20°C -12 to -7, peroxides < 20 mmol/L, and percentages corresponding to the area percentage of the peaks corresponding to the chemical compounds of interest relative to the total area of the peaks in the chromatogram obtained by GC/FID analysis on a polar column: limonene 0%-0.5%, eucalyptol 0%-1 %, cis-beta-ocimene 4%-10%, trans-beta-ocimene 1.5%-6%, 3-octanone 0%-2%, p-cymene 0%-0.6%, camphor 0-0.5%, linalool 25%-32%, linalyle acetate 32%-42%, beta-caryophyllene 3%-6%, 4-terpinenol 2%-6%, lavandulyle acetate 2%-6%, lavandulol 0.3%-1.5%.

Supplementary Figures

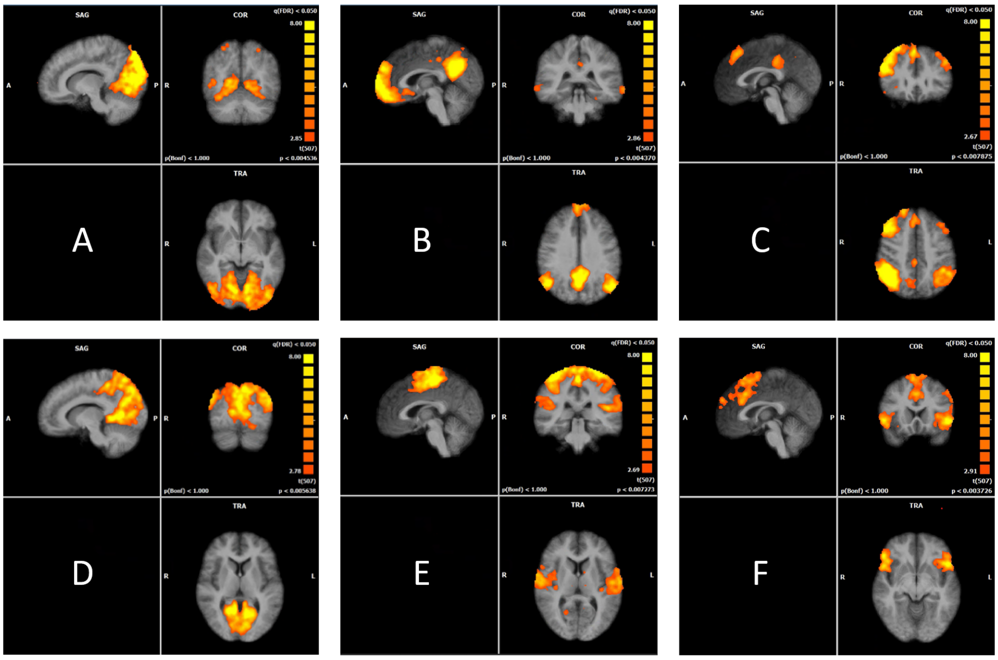

Supplementary Figure 1: Six major resting state networks detected within the group level analysis of the ICA data, using the Lavender condition during odour exposure. Each map depicts the results of a second level statistic based on the sorted group components resulting from the group ICA and are thresholded using the False Discovery Rate (FDR) method (Genovese et al 2002). The following networks were identified: A) Lateral Visual, B) Default Mode (DMN), C) Fronto-Parietal, D) Medial Visual, E) Motor, F) Salience.

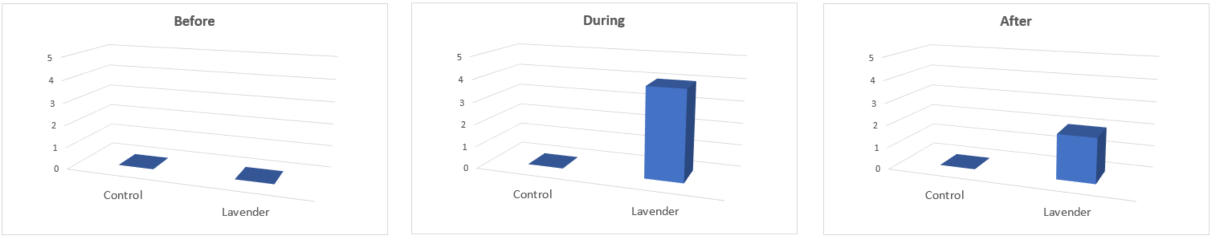

Supplementary Figure 2: The basic expectation about the difference in relevant resting state networks between Lavender and Control conditions at three time points. In line with our expectations, no group effect was found in the before condition, a large effect was found during the odour application which persisted at a smaller amplitude in the after condition.

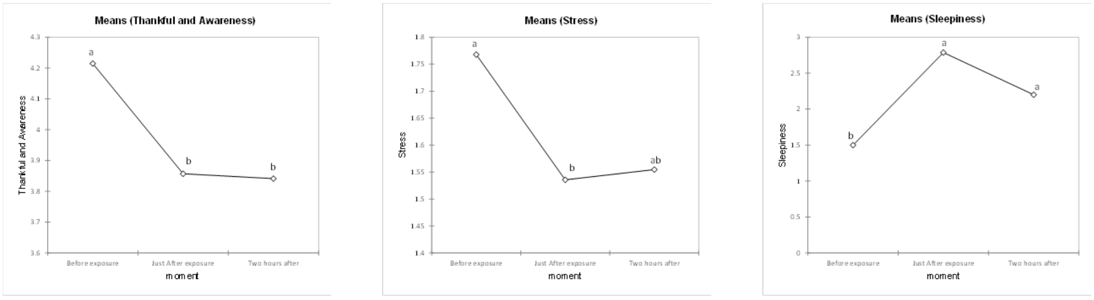

Supplementary Figure 3**:** Subscores of the Smith Relaxation Survey inventory for the dimensions “Thankfulness and Awareness”, “Stress” and “Sleepiness”. A significant decrease in Thankfulness and Awareness dimension was observed post-odour exposure compared to pre-exposure, with no significant difference between the one-and two-hour conditions. A trend for a significant decrease at one and two hours after was observed for the dimension Stress (10% level). Sleepiness increased and remained significantly higher compared to the pre-exposure condition for both the immediate post-exposure and two-hour post-exposure conditions.

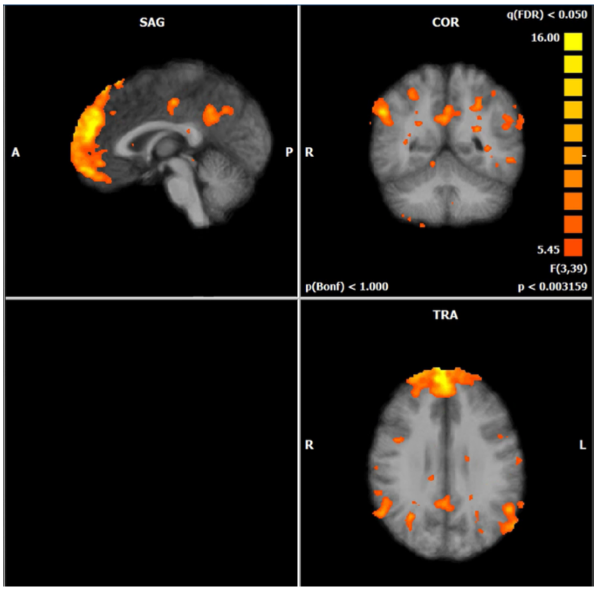

Supplementary Figure 4: Interaction between Odour and Time point of the ANOVA with only four levels for the factor time point.
